# Predictive value of delta radiomics in xerostomia after chemoradiotherapy in patients with stage III-IV nasopharyngeal carcinoma

**DOI:** 10.1186/s13014-024-02417-6

**Published:** 2024-02-28

**Authors:** Mengze Wang, Yuzhen Xi, Luoyu Wang, Haonan Chen, Feng Jiang, Zhongxiang Ding

**Affiliations:** 1 Department of Radiology, Zhejiang Cancer Hospital, Hangzhou, China; 2 Department of Radiology, 903 RD Hospital of PLA, Hangzhou, China; 3 Department of Radiology, Hangzhou First People’s Hospital, Hangzhou, China; 4 Department of Radiology, Zhejiang Hospital, Hangzhou, China; 5 Department of Head and Neck Radiotherapy, Zhejiang Province Key Laboratory of Radiation Oncology, Zhejiang Cancer Hospital, Hangzhou, China

**Keywords:** Nasopharyngeal carcinoma, Magnetic resonance imaging, Delta Radiomics, Parotid gland injury, Xerostomia

## Abstract

**Background:**

Xerostomia is one of the most common side effects in nasopharyngeal carcinoma (NPC) patients after chemoradiotherapy. To establish a Delta radiomics model for predicting xerostomia secondary to chemoradiotherapy for NPC based on magnetic resonance T1-weighted imaging (T1WI) sequence and evaluate its diagnostic efficacy.

**Methods:**

Clinical data and Magnetic resonance imaging (MRI) data before treatment and after induction chemotherapy (IC) of 255 NPC patients with stage III-IV were collected retrospectively. Within one week after CCRT, the patients were divided into mild (92 cases) and severe (163 cases) according to the grade of xerostomia. Parotid glands in T1WI sequence images before and after IC were delineated as regions of interest for radiomics feature extraction, and Delta radiomics feature values were calculated. Univariate logistic analysis, correlation, and Gradient Boosting Decision Tree (GBDT) methods were applied to reduce the dimension, select the best radiomics features, and establish pretreatment, post-IC, and Delta radiomics xerostomia grading predictive models. The receiver operating characteristic (ROC) curve and decision curve were drawn to evaluate the predictive efficacy of different models.

**Results:**

Finally, 15, 10, and 12 optimal features were selected from pretreatment, post-IC, and Delta radiomics features, respectively, and a xerostomia prediction model was constructed with AUC values of 0.738, 0.751, and 0.843 in the training set, respectively. Only age was statistically significant in the clinical data of both groups (*P* < 0.05).

**Conclusion:**

Delta radiomics can predict the degree of xerostomia after chemoradiotherapy for NPC patients and it has certain guiding significance for clinical early intervention measures.

**Supplementary Information:**

The online version contains supplementary material available at 10.1186/s13014-024-02417-6.

## Background

Nasopharyngeal carcinoma (NPC) is a malignant tumor arising from the epithelium of the nasopharyngeal mucosa. Most NPC cells are highly sensitive to ionizing radiation, and hence radiation therapy is currently the treatment of choice for NPC patients [1–2]. Due to the limitation of the anatomical location of NPC, the radiation field area often involves the surrounding normal tissues. The radical dose exceeds the tumor tolerance; thus, the radiotherapy-induced damage to the tissues and organs in the radiation area is extensive and lasting [3]. Parapharyngeal space is the most common site of lymph node metastasis, and the parotid gland is adjacent to the parapharyngeal space, rendering it inevitable to be exposed to high-doses radiation during radiotherapy and one of the organs at risk of radiotherapy in NPC patients. Nonetheless, the three-dimensional conformal and intensity-modulated radiotherapy techniques have advanced and are widely used. Although the survival prognosis of NPC patients has improved significantly [4–5], xerostomia caused by parotid gland injury is still one of the most common long-term adverse reactions after radiotherapy for NPC, which has severely affected the quality of life of patients, including speech, swallowing, and overall oral problems [6–7].

Magnetic resonance imaging (MRI) is the primary imaging modality and diagnostic tool for NPC patients, routinely used for the development of radiotherapy plans and the evaluation of treatment efficacy. Previous studies have found that MRI can evaluate the morphological changes, volume reduction and signal intensity changes of parotid gland after radiotherapy [8–9], but traditional imaging techniques have limitations. As a rapidly developing disease diagnosis and auxiliary detection technology, radiomics can quantify tissue heterogeneity and reveal texture features that cannot be fully recognized by qualitative MRI analysis. It has high clinical value in tumor staging [10], differential diagnosis [11], efficacy evaluation [12] and prognosis prediction [13] of nasopharyngeal carcinoma. It can also provide imaging biomarkers for radiotherapy-related side effects [14–17]. In addition, radiomics has been applied to related studies of parotid gland. For example, MRI radiomics model based on texture analysis of T2 weighted sequence has improved the diagnostic performance of non-subspecialty radiologist for the differential diagnosis between pleomorphic adenoma and Warthin tumor [18]. Therefore, we hypothesize that radiomics analysis of the parotid gland can provide early prediction of xerostomia after chemoradiotherapy. Delta radiomics is a new form of radiomics, an analysis of changes in the features at different acquisition time points that can longitudinally reflect the treatment-induced changes. A previous study has combined the CT imaging changes of parotid gland and salivary volume during radiotherapy to predict radiation-induced acute thirst levels early [19]. However, as far as we know, there are few MRI-based Delta radiomics studies on xerostomia after chemoradiotherapy. The purpose of this study was to establish traditional radiomics model and Delta radiomics model based on MRI before treatment and after induction chemotherapy and observe the heterogeneity of the parotid gland, aiming to provide novel ideas for the early clinical prediction of xerostomia after chemoradiotherapy for NPC patients.

## Materials and methods

### Study subjects and groups

**Fig. 1:**
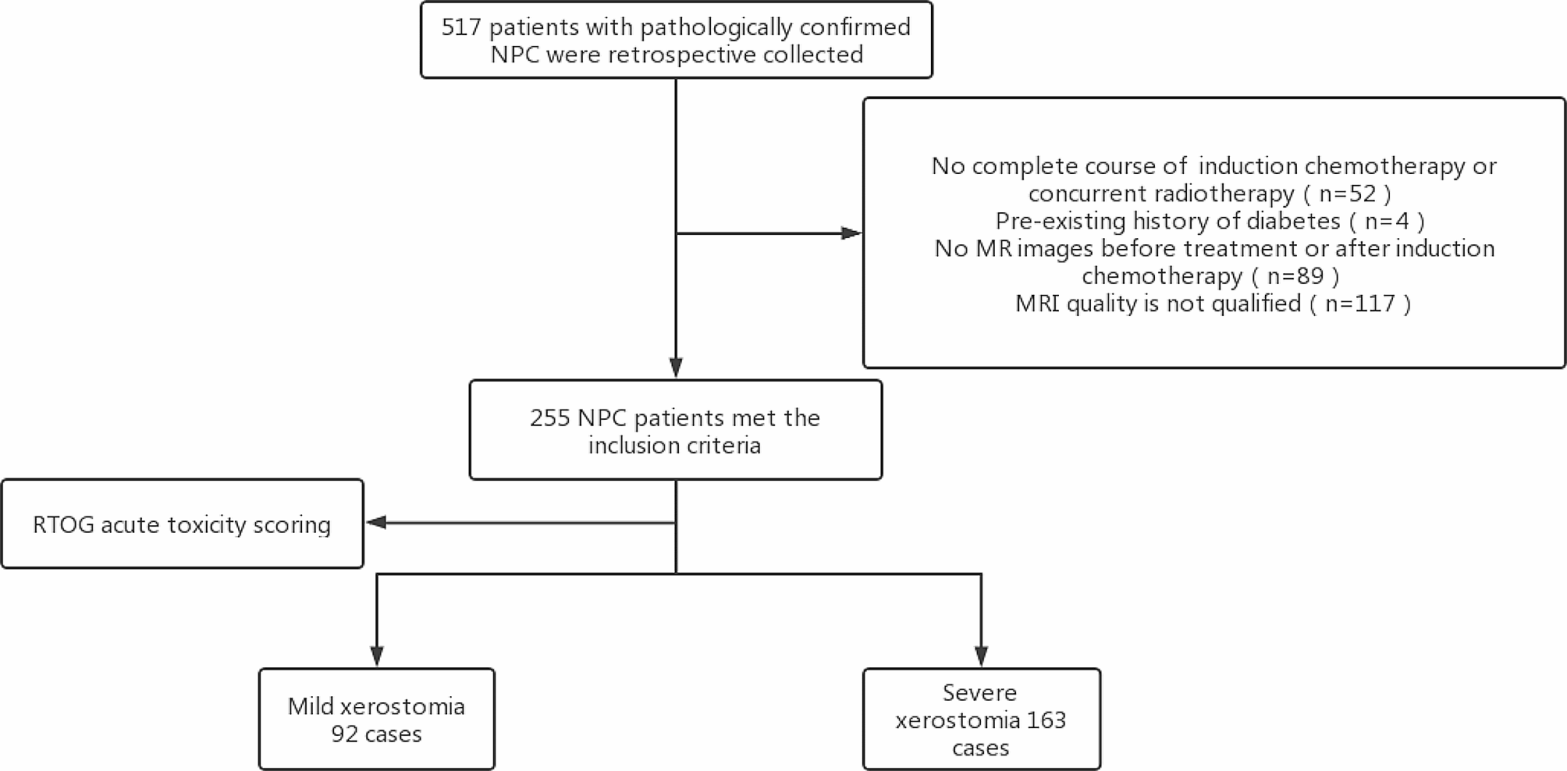
Case screening process. A total of 255 eligible cases were screened, including 92 patients with mild and 163 patients with severe xerostomia

This study was approved by the Scientific Research Ethics Committee of the Cancer Hospital of the University of Chinese Academy of Sciences (Zhejiang Cancer Hospital) and Affiliated Hangzhou First People’s Hospital, Zhejiang University School of Medicine. Because it is a retrospective study, patient consent is waived. The data of 517 primary NPC patients in the Zhejiang Cancer Hospital from 2012 to 2016 were collected; the inclusion criteria were as follows: (1) patients with pathologically confirmed newly diagnosed NPC; (2) patients who received a complete course of concurrent chemoradiotherapy (CCRT) or radiotherapy after 1–3 cycles of induction chemotherapy (IC); (3) MRI examinations were conducted before and after IC. The exclusion criteria were as follows: (1) previous or current salivary gland-related diseases and surgery; (2) history of xerostomia symptoms, such as diabetes and diabetes insipidus; (3) history of head and neck radiotherapy; (4) data with incomplete MR images and unqualified image quality were excluded. The patient selection process is illustrated in Fig. 1. All patients were treated with intensity-modulated radiotherapy, with radiation doses ranging from 66.0 to 71.0 Gy for primary gross tumor volume of NPC (PGTVnx) and 63.0–72.1 Gy for gross tumor volume of cervical lymph nodes (GTVnd) in 30–32 fractions.

Herein, we used the Chinese version of the European Organisation for.

Research and Treatment of Cancer (EORTC) Quality of Life Scale QLQ-HampN35 (V1.0) to assess the degree of xerostomia in NPC patients after chemoradiotherapy within one week. According to the acute radiation injury grading of the radiation therapy oncology group (RTOG), the patient’s salivary gland injury was categorized into: Grade 0: no change from baseline; Grade 1: mild mouth dryness/slightly thickened saliva/may have slightly altered taste such as metallic taste/these changes not reflected in alteration in baseline feeding behavior, such as increased use of liquids with meals; Grade 2: moderate to complete dryness/thick, sticky saliva/markedly altered taste; Grade 3: severe xerostomia, no irritation, often waking up at night to drink water; Grade 4: acute salivary gland necrosis [20]. The salivary gland injury ≥ grade 2 was defined as severe xerostomia, while grade 0 or 1 indicated mild xerostomia. The enrolled patients included nine factors of the clinical data, including gender, age, T stage, N stage, induction chemotherapy course, induction chemotherapy regimen, CCRT course, and dose (PGTVnx and GTVnd).

### MRI examination method

MR images were obtained from all patients using two different MRI scanners (Siemens Magnetom Symphony 1.5T and Siemens SKyra 3.0T, Munich, Germany) before treatment and after IC, with an interval of 1–2 months. Standard head coil was used for scanning, Conventional MRI scans T1-weighted imaging (T1WI) were obtained at the following parameters: (1) 3.0T MRI scan parameters: transverse T1WI repetition time (TR) 1800 ms, echo time (TE) 9.4 ms, 320*75 matrix, 1.0*0.7*5.0 mm; (2) 1.5T MRI scan parameters: transverse T1WI TR 380 ms, TE 7.0 ms, 304*80 matrix, 1.07*0.86*5.0 mm. The remaining parameters were consistent for both instruments: slice thickness 5 mm, interslice distance 5 mm, field of view (FOV) = 240 × 230 mm, 90° flip angle.

### Image segmentation

Each patient’s head and neck MRI examinations before and after induction chemotherapy were retrieved from the hospital’s PACS system, and the DICOM format images of all sequences were exported. The ITK-SNAP software (Fig. www.itksnap.org, Version3.8.0) imports the images and selects the transaxial T1WI sequence to store it as the original image. Since parotid segmentation needs to be repeated at different time points and manual segmentation is time-consuming, we developed a deep learning model for semi-automated segmentation of the parotid regions for efficient segmentation. The parotid gland was manually segmented layer-by-layer on the T1WI sequence before treatment, then stored as regions of interest (ROIs). A deep learning model was established on the U^2^ Netp network using the segmentation results before treatment as a reference (see the Supplementary Materials for specific methods) to segment the images after IC, followed by a further manual correction to obtain semi-automatic segmentation results. The final segmentation results were also verified by a senior head and neck radiologist.

### Feature extraction and delta feature calculation

1. Loading data and adding labels: First, all the original images and ROI data were imported into the joint shadow u AI Research Portal (Version 1.4) software [21]; Add Labels was selected to add labels to the subjects (set patients with severe xerostomia marked as “1”; patients with mild xerostomia, marked as “0”).
2. MRI preprocessing: Resampling and normalization of the original data with the following parameters: Interpolator = NearestNeighbor, resampled Pixel Spacing = 1*1*1, bin Width = 25, label = 1, normalize Scale = 1, minimum ROI Dimensions = 2, minimum ROI Size = None, voxel Based = False. Wavelet = coif1, level = 1.
3. Feature extraction: First select the following features: First Order, shape features (Shape), gray level co-occurrence matrix (GLCM), gray level dependence matrix (GLDM), gray level size zone matrix (GLSZM), gray level run length matrix (GLRLM), and neighboring gray-tone difference matrix (NGTDM). Then select the filter type as follows: Original, BoxMean, AdditiveGaussianNoise, BinomialBlurImage, BinomialBlurImage, BoxsigmaImage, LoG, wavelet, Normalize, LaplacianSharpening, DiscreteGaussian, Mean, SpeckleNoise, RecursiveGaussian, and ShotNoise. Finally, the radiomics profiles for 255 patients before treatment and after IC were extracted.
4. For Delta radiology profile estimation, the change in each radiology profile was calculated using the following equation:
  Delta feature value = (Feature value 2 - Feature value 1).
  Herein, feature value 2 represents the post-IC MRI value, and feature value 1 represents the pretreatment MRI value. This formula has been applied previously for calculating the Delta feature value [12].

### Feature selection, model establishment, and Evaluation

**Fig. 2:**
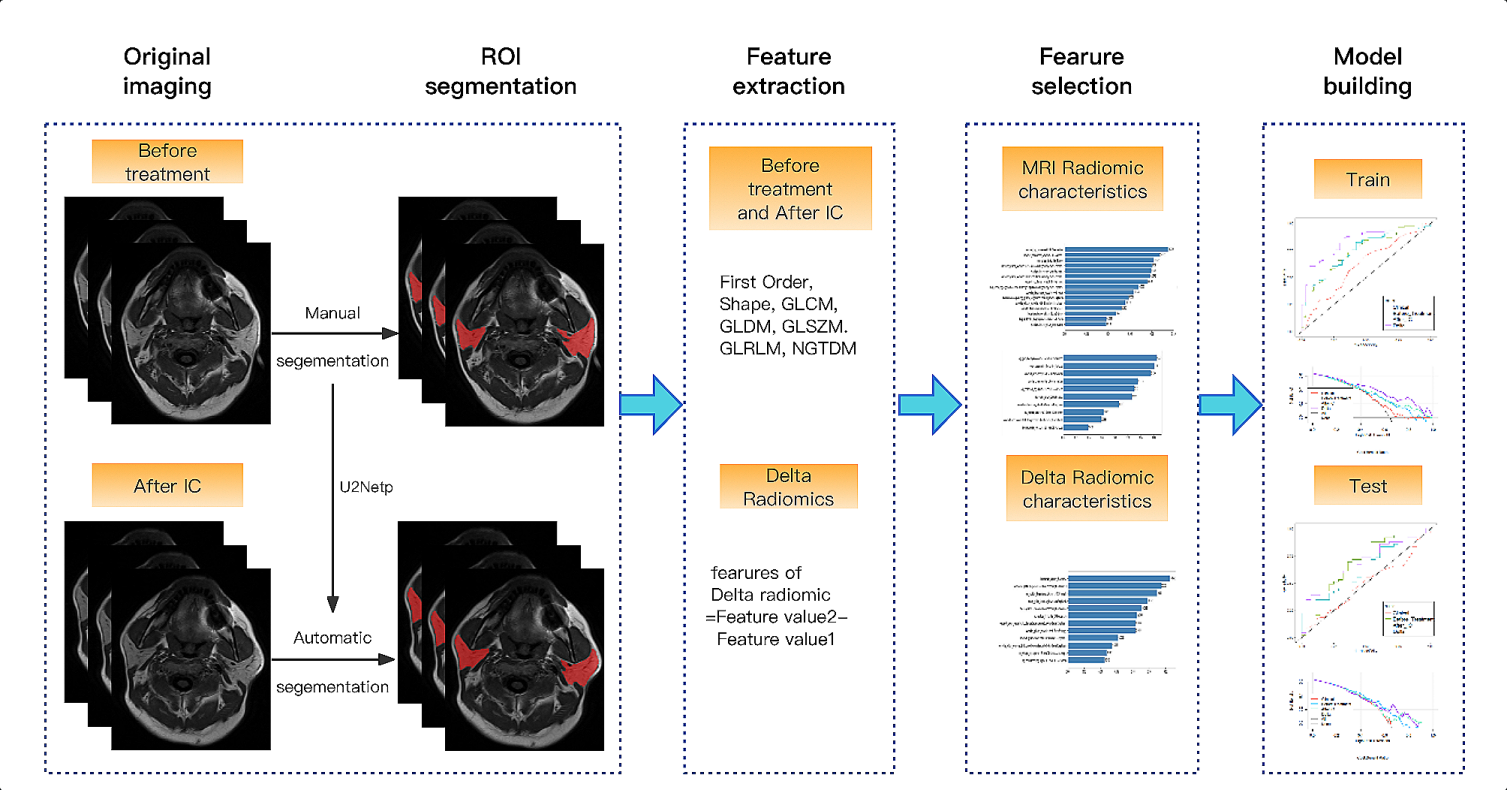
Flow chart of radiomics: feature value 2 represents radiomics feature value after IC, feature value 1 represents radiomics feature value before treatment

Statistical analyses were carried out using R 3.5.1 and Python 3.5.6 software. *P* < 0.05 indicating statistical significance. All patients were randomly were randomly divided into training, and test sets at a ratio of 7:3, and the data were normalized. Pretreatment radiomics features, post-IC radiomics features, and Delta features were sequentially screened using univariate logistic analysis, correlation, and GBDT methods to select the optimal feature subset. Logistic regression models were built based on the optimal feature subset of the training set. The receiver operating characteristic (ROC) curve was used to evaluate the performance of the machine learning model. The area under curve (AUC), diagnostic accuracy (ACC), sensitivity (SENS), specificity (SPEC), F1 score, positive predictive value (PPV), and negative predictive value (NPV) of the training and the test sets were calculated, respectively. The flow chart of the radiomics is illustrated in Fig. 2.

### Statistical methods of clinical data

Statistical analyses were conducted using SPSS 23.0 software. Nine clinical factors in 255 patients were first included in the influencing factors, which were then divided into two groups according to mild and severe xerostomia, followed by a comparison between the two groups. Data that fit a normal distribution were presented as mean and standard deviation, while data that were not normally distributed were presented as medians and interquartile ranges. Continuous variables were tested for normality. T-test was used to compare the normal distribution variable, Mann–Whitney U test for non-normal distribution, and chi-square test for categorical variables. *P* < 0.05 indicated a statistical significance.

## Results

### Comparison of clinical baseline data between groups

A total of 255 patients were enrolled and divided into mild (*n* = 92) and severe (*n* = 163) groups according to the degree of xerostomia. Age differed significantly in the clinical data of the two groups (*P* < 0.05), but not gender, IC course, CCRT course, IC regimen, T stage, N stage, and dose (PGTVnx and GTVnd) (Table 1).

**Table 1:** Comparison of clinical baseline data between mild and severe xerostomia groups

Clinical		Mild xerostomia (*n* = 92)	Severe xerostomia (*n* = 163)	*P*
Sex	Man	67 (72.8%)	109 (66.9%)	> 0.05
	Woman	25 (27.2%)	54 (33.1%)	
Age (years)		48.11 ± 9.58	50.64 ± 9.62	< 0.05
Induction chemotherapy course	1	1	1	> 0.05
	2	2	4	
	3	89	158	
Concurrent chemotherapy course	0	2	3	> 0.05
	1	6	10	
	2	84	150	
Chemotherapy protocol	1	41	86	> 0.05
	2	51	77	
T stage	1	3	6	> 0.05
	2	7	14	
	3	58	95	
	4	24	48	
*N* stage	0	4	4	> 0.05
	1	34	71	
	2	44	71	
	3	10	17	
Stage	III	59	104	> 0.05
	IV	33	59	
Dose	PGTVnx (Gy)	69.59 ± 0.98	69.75 ± 0.92	> 0.05
	GTVnd (Gy)	67.53 ± 2.00	67.21 ± 5.24	> 0.05

### Radiomics feature selection results

**Fig. 3:**
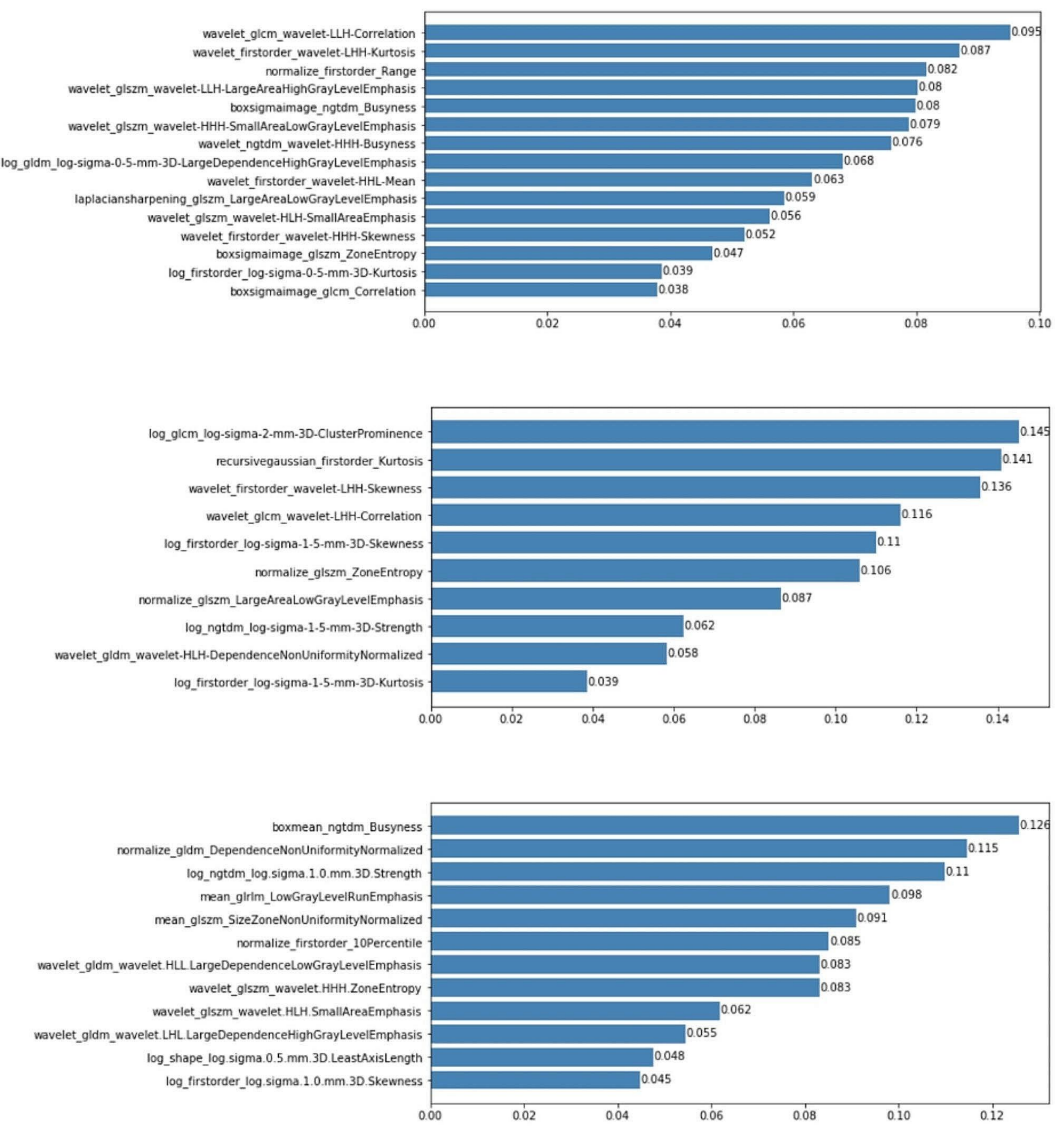
15, 10, and 12 optimal subsets selected in pretreatment **(A)**, after IC **(B)**, and Delta **(C)** radiomics features, respectively

A total of 2600 filtered features were extracted from T1WI sequences before and after IC, and Delta features were assessed. After univariate logistic analysis, the number of features remaining before treatment, after IC, and Delta features were 141, 133, and 159, respectively. After correlation analysis and screening, the number of remaining features was 34, 23, and 25, respectively. Finally, the GBDT method was used to select 15, 10, and 12 optimal radiomics features, respectively. Among these, the optimal subset before treatment included first-order features (5) and texture features (10), the optimal subset after IC included first-order features (4) and texture features (6), and the optimal subset of Delta included first-order features (2), shape features (1) and texture features (9), as shown in Fig. 3.

### Model predictive power analysis

**Fig. 4:**
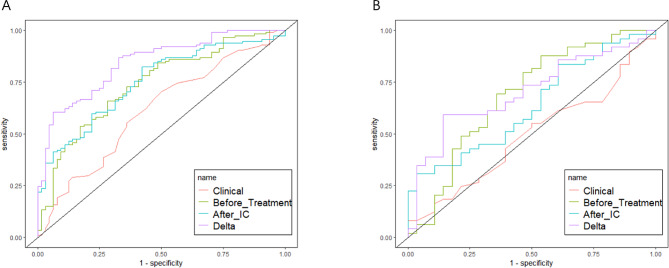
ROC curves for the clinical model, the traditional radiomics model of before treatment and after IC, and the Delta radiomics model in the training set **(A)** and the test set **(B)**

**Fig. 5:**
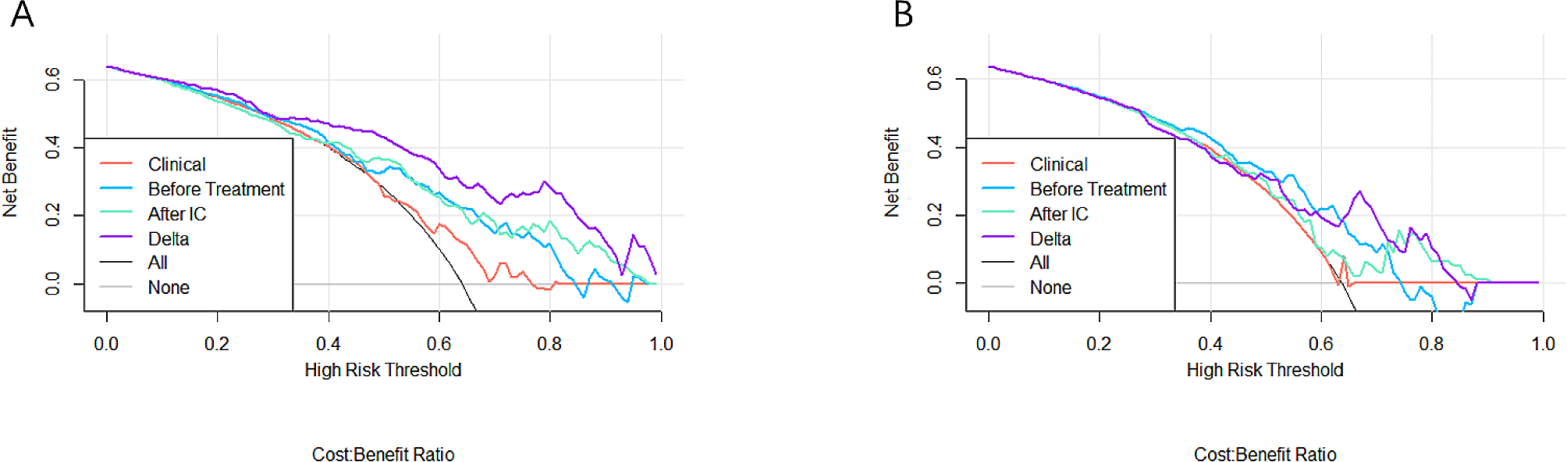
Decision curves of training set **(A)** and test set **(B)**. The X-axis represents the model power, ranging from 0–100%, the Y-axis measures the net benefit, and purple, green, blue, and orange represent the decision curves of different models, respectively. None and ALL are two reference lines; the closer the other model curves are to the two reference lines, the less value is applied; the high ordinate in the case of the same abscissa represents the better model efficacy

The AUC values, ACC, SENS, SPEC, F1 score, PPV, and NPV of the pretreatment, post-IC, and Delta radiomics logistic regression models in the training and test sets, respectively, are summarized in Table 2. ROC curves and Decision curves of the training and test sets of all models are shown in Fig. 4 and Fig. 5. All three models have certain predictive value, while Delta radiomics model has better predictive performance compared to both parameters.

**Table 2:** Verification results corresponding to the training and test sets

		AUC	ACC	SENS	SPEC	F1	NPV	PPV
Before treatment	Training set	0.738	0.674	0.649	0.719	0.718	0.535	0.804
	Test set	0.684	0.636	0.612	0.679	0.682	0.500	0.769
After IC	Training set	0.751	0.730	0.816	0.578	0.795	0.638	0.775
	Test set	0.635	0.610	0.714	0.429	0.700	0.462	0.686
Delta	Training set	0.843	0.719	0.596	0.938	0.731	0.566	0.944
	Test set	0.702	0.649	0.531	0.857	0.658	0.511	0.867

Note: AUC, area under ROC curve; ACC, accuracy; SENS, sensitivity; SPEC, specificity; PPV, positive predictive value; NPV, negative predictive value

## Discussion

In this study, the MR images before and after IC were used to extract radiomics features, and the change in radiomics features before and after chemotherapy was calculated. After three feature selections, two traditional radiomics and one Delta dynamic radiomics models were constructed to predict the severity of xerostomia after chemoradiotherapy for NPC patients. Delta radiomics models had a better predictive performance compared to the two traditional radiomics models.

In recent years, the increasing survival of NPC patients has raised the demand of the patients for quality of life. Therefore, it is of great clinical significance to make early predictions of xerostomia after chemoradiotherapy in NPC patients, timely adjust the treatment regimen, and minimize parotid gland function impairment [4]. Both conventional xerostomia assessment systems [22] and traditional imaging [23–24] studies are based on subjective assessment and measurement, lacking accurate quantification. A traditional radiomics studies based on imaging biomarkers of CT [14–15], MR [16], or positron emission tomography (PET) [17] can predict late xerostomia after radiotherapy, while some studies have used texture analysis to evaluate the structural changes in the parotid gland tissue during radiotherapy and established prediction models for acute xerostomia after radiotherapy [25]. Most of these studies are based on the features of static radiomics; however, the course of treatment and the occurrence and development of xerostomia are dynamic. Interestingly, dynamic radiomics studies are associated with side effects after radiotherapy for NPC patients, except for predicting radiation-induced temporal lobe injury based on MR images [26]; most of these use CT images [19, 27]. Repeated use of CT examination may cause ionizing radiation hazard to human body while we use the most commonly used images of conventional MRI examination, and MRI has obvious advantages in soft tissue resolution compared to CT and PET images. In addition, the current study predicted chemoradiotherapy-induced xerostomia using images before treatment and after IC, i.e., images before radiotherapy.

There are two common formulas for calculating delta feature value, one of which is 100 x (post-treatment feature value - baseline feature value)/baseline feature value [28], and the other is Delta feature value = (Feature value 2 - Feature value 1), the latter is adopted in this study. After dimensionality reduction of radiomics features the best radiomics feature sets were selected: including 15 before treatment, 10 after IC, and 12 Delta radiomics features, and prediction models were established respectively. Among these, Delta radiomics model has the best predictive power, and the five features with the highest coefficients among Delta radiomics features are texture features: ngtdmBusyness, gldmDependenceNonUniformityNormalized (DNN), ngtdm_log.sigma.1.0.mm 3D.Strength, glrlmLowGrayLevelRunEmphasis (LGLRE), and glszmSizeZoneNonUniformityNormalized (SZNN). NGTDM represents the gray-tone difference between a voxel and its neighbors, wherein high values of busy indicate rapid changes in the intensity around local voxels. Strength indicates the significance and uniqueness of changes around the voxel, an image with slow change in intensity but large differences in gray level intensities. DNN measures the similarity of dependencies in images, with lower values indicating a high homogeneity of dependency in parotid images. SZNN measures the variability of the volume of the size zone volumes throughout the image, with lower values indicating a high homogeneity among zone size volumes in the image [29]. LGLRE measures the distribution of low gray-level values with high values indicating a significant proportion of low gray-level values in parotid images. Similar to previous reports, the current study suggested that patients who develop xerostomia may have heterogeneous salivary gland tissue [14, 30]. In addition, we found that variations around voxels may be associated with tolerance of the parotid gland following chemoradiation. The three features with the highest coefficients among pretreatment radiomics features were glcm-Correlation, firstorderLHH-Kurtosis, and firstorderRange, wherein correlations represent the roughness of the image texture. Kurtosis and Range belong to the first-order feature; Kurtosis is a measure of the “peak” of the signal intensity distribution in the image ROI, while Range represents the range of gray values in the ROI. The three features with the highest coefficients among the radiomics features after induction chemotherapy were glcm-sigma-2-mm-3D-ClusterProminence, firstorderKurtosis, and firstorder-LHH-Skewness. Cluster prominence and skewness measure the asymmetry of the distribution of values about the mean value; higher values represent asymmetry around the mean. In addition, We found that these three radiomics models have two common features, namely glszm_ZoneEntropy and firstorder_Skewness. ZoneEntropy measures the uncertainty/randomness in the distribution of zone sizes and gray levels. The higher the value, the more heterogeneous the texture pattern. we also found that the first-order features accounted for about 66.7% of the three features with the highest coefficients of the two traditional radiomics features, while Delta radiomics features were concentrated in the high-order features and had the characteristics of accurate quantification images, further indicating that Delta radiomics model has the best prediction effect.

### Limitations

The limitations of this study are as follows: Firstly, the study was retrospective, and hence, a selection bias is inevitable. Secondly, only a single T1WI sequence was used for prediction, and multisequence combination would be used for subsequent studies. Thirdly, we conducted only a short-term xerostomia study. Chemoradiotherapy may have a long-term effect on parotid gland function in NPC patients; thus, the dynamic changes of xerostomia after chemoradiotherapy will be considered for future studies.

## Conclusion

In conclusion, Delta radiomics based on MR images has high predictive power in predicting xerostomia caused by parotid gland injury after chemoradiotherapy for NPC patients and it has certain guiding significance for clinical early intervention measures.

### Electronic supplementary material

Below is the link to the electronic supplementary material.

Supplementary Material 1

## Data Availability

The datasets used and analyzed during the current study are available from the corresponding author on reasonable request.

## Abbreviations

NPC: Nasopharyngeal carcinoma
T1WI: T1-weighted imaging
MRI: Magnetic resonance imaging
IC: nduction chemotherapy
CCRT: concurrent chemoradiotherapy
GBDT: Gradient Boosting Decision Tree
ROC: receiver operating characteristic
CT: computed tomography
MR: magnetic resonance
EORTC: European Organisation for Research and Treatment of Cancer
RTOG: radiation therapy oncology group
PGTVnx: primary gross tumor volume of nasopharynx
GTVnd: gross tumor volume of cervical lymph nodes
TR: repetition time
TE: echo time
FOV: field of view
ROIs: regions of interest
GLCM: gray level co-occurrence matrix
GLDM: gray level dependence matrix
GLSZM: gray level size zone matrix
GLRLM: gray level run length matrix
NGTDM: neighboring gray-tone difference matrix
AUC: area under curve
ACC: diagnostic accuracy
SENS: sensitivity
SPEC: specificity
PPV: positive predictive value
NPV: negative predictive value
PET: positron emission tomography
DNN: DependenceNonUniformityNormalized
LGLRE: LowGrayLevelRunEmphasis
SZNN: SizeZoneNonUniformityNormalized
