# Supplementary material for: Predictive value of delta radiomics in xerostomia after chemoradiotherapy in patients with stage III-IV nasopharyngeal carcinoma
Source: Radiat Oncol. 2024 Feb 28;19:26. doi: 10.1186/s13014-024-02417-6 (PMC10900635; doi:10.1186/s13014-024-02417-6)
Supplement: Supplementary file 1 — Supplementary Material 1 [file 13014_2024_2417_MOESM1_ESM.docx]

Supplementary materia

U^2^-Net is a simple yet powerful deep network architecture, and U2 -Net has been verifed useful in image segmentation task ^[1, 2]^. Our study specifically operates using the following link: https://github.com/xuebinqin/U-2-Net.

1. Pan B, Qi N, Meng Q, Wang J, Peng S, Qi C, Gong NJ, Zhao J. Ultra high speed SPECT bone imaging enabled by a deep learning enhancement method: a proof of concept. EJNMMI Phys. 2022;9(1):43.
2. Qin X, Zhang Z, Huang C, Dehghan M, Zaiane OR, Jagersand M. U2-Net: going deeper with nested U-structure for salient object detection. Pattern Recognit. 2020;106: 107404.
